# Machine learning analysis of PM1 impact on visibility with comprehensive sensitivity evaluation of concentration, composition, and meteorological factors

**DOI:** 10.1038/s41598-024-67576-8

**Published:** 2024-07-20

**Authors:** Grzegorz Majewski, Bartosz Szeląg, Wioletta Rogula-Kozłowska, Patrycja Rogula-Kopiec, Andrzej Brandyk, Justyna Rybak, Maja Radziemska, Ernesta Liniauskiene, Barbara Klik

**Affiliations:** 1 Institute of Environmental Engineering, Warsaw University of Life Sciences, 02-776 Warsaw, Poland; 2 Faculty of Fire Safety Engineering, Fire University, 01-629 Warsaw, Poland; 3 Institute of Environmental Engineering, Polish Academy of Sciences, 41-819 Zabrze, Poland; 4 Faculty of Environmental Engineering, Wrocław University of Science and Technology, 50-370 Wrocław, Poland; 5 Department of Hydrotechnical Engineering, Faculty Environmental Engineering, Kaunas Forestry and Environmental Engineering University of Applied Sciences, 53101 Girionys, Kaunas Lithuania

**Keywords:** Atmospheric chemistry, Environmental impact, Atmospheric science, Atmospheric dynamics, Ecological modelling

## Abstract

This study introduces a novel approach to visibility modelling, focusing on PM1 concentration, its chemical composition, and meteorological conditions in two distinct Polish cities, Zabrze and Warsaw. The analysis incorporates PM1 concentration measurements as well as its chemical composition and meteorological parameters, including visibility data collected during summer and winter measurement campaigns (120 samples in each city). The developed calculation procedure encompasses several key steps: formulating a visibility prediction model through machine learning, identifying data in clusters using unsupervised learning methods, and conducting global sensitivity analysis for each cluster. The multi-layer perceptron methods developed demonstrate high accuracy in predicting visibility, with R values of 0.90 for Warsaw and an RMSE of 1.52 km for Zabrze. Key findings reveal that air temperature and relative humidity significantly impact visibility, alongside PM1 concentration and specific heavy metals such as Rb, Vi, and Cd in Warsaw and Cr, Vi, and Mo in Zabrze. Cluster analysis underscores the localized and complex nature of visibility determinants, highlighting the substantial but previously underappreciated role of heavy metals. Integrating the k-means clustering and GSA methods emerges as a powerful tool for unravelling complex mechanisms of chemical compound changes in particulate matter and air, significantly influencing visibility development.

## Introduction

Visibility (Vis) stands as a straightforward indicator of air quality, as confirmed by multiple research studies^1,2^. Defined as the degree of atmospheric clarity over the maximum horizontal distance observable, this parameter reliably measures changes in air quality^3,4^. For instance, Doyle and Dorling^5^ demonstrated that Vis trends in the UK over more than 40 years strongly correlated with changes in particulate matter (PM) concentrations, indicating variations in air quality. Chang et al.^6^ observed similar trends in six megacities in China, where rapid urbanization and industrialization significantly impacted Vis, reflecting the deteriorating air quality. Zhao et al.^7^ highlighted that in Northeast China, Vis impairment directly correlated with levels of particulate matter, serving as a practical measure of air pollution levels. Singh et al.^8^ analyzed long-term UK Vis data, showing how meteorological conditions and atmospheric pollutants collectively affected Vis, underscoring its role as an air quality indicator. Previous research revealed that the characteristics of physical and chemical properties of aerosols, namely their concentration^9^, chemical composition^10,11^, and particle size distribution^12,13^, exert a decisive influence over Vis behaviour^9–11^. This influence becomes particularly pronounced during autumn and winter, attributed to unfavourable meteorological conditions^12^. Moreover, key particle components, such as (NH_4_)_2_SO_4_, NH_4_NO_3_, and organic matter (OM), play a substantial role in Vis impairment due to their potent light-scattering effects^13^. Importantly, it’s observed that particles in the accumulation mode have a more pronounced impact on Vis compared to those in the coarse mode unless influenced by specific weather phenomena like sandstorms or fog. Simultaneously, meteorological factors, with relative humidity (RH) at the forefront alongside wind speed (windS) and regional pollutant transport, emerge as critical contributors to Vis degradation^14^. Elevated RH levels contribute to particle size growth, a result of their hygroscopic properties, influencing radiative forcing and causing fluctuations in Vis^15,16^. For instance, sulfate, nitrate, and ammonium salts of particles are identified as potent hygroscopic species^17^. Furthermore, increased RH fosters gas-to-particle conversion processes of precursor gases, fostering the formation and accumulation of secondary sulfate and nitrate, thereby exacerbating Vis impairment^18^.

Elevated aerosol concentrations, coupled with their significant hygroscopic growth, contribute substantially to Vis deterioration, particularly on hazy days^19^. Moreover, this phenomenon can extend to fog formation, triggered by the activation of cloud condensation nuclei when RH exceeds 100%. The interplay becomes more intricate as Vis responds exponentially to changes in PM2.5 mass concentration when RH is below 80%. Contrastingly, with RH at or above 80%, the hygroscopic growth of particles takes precedence in diminishing Vis, as highlighted by Gultepe et al.^20^. In conditions of high RH, the aerosol liquid water content emerges as a critical factor. It not only influences aerosol mass loading, light extinction, and Vis^21^ but also serves as a medium for multiphase chemical reactions in the ambient environment. This, in turn, enhances the processes of secondary inorganic aerosol formation^22^.

Advanced numerical modelling techniques^23,24^ alongside statistical and empirical methods^1,18,25–31^ have been instrumental in predicting changes in Vis. A comprehensive summary of various statistical approaches used for Vis prediction is outlined in Table 1.

**Table 1:** Compilation of sample statistical approaches to predicting visibility.

Study	Region	Input	Output	Model/analysis
Dao et al.^30^	28 City (China)	PM2.5, OC, EC, x, y	Vis	MPR
Zhao et al.^29^	Mount Tai (China)	PM1(2.5), RH	Vis	MPR
Li et al.^54^	Irak, Kuwait	AOD, H, T, PM, t, sesonality, Elev	Vis	RF
		NDVI, lU, Traffic		
Deng et al.^59^	5 City: Xiamen, Fuzhou, Taipei, Taichung, Tainan	T(R = 0.51–0.77), RH(R = 0.45),		
		Press(R = − 0.49–0.70), NO_2_(R = − 0.62–0.81)	Vis	Correlation
		SO_2_(R = − 0.58 to − 0.67, 0.43), PM10(R = − 0.58–0.66)		
		PM2.5(R = − 0.68 to − 0.76, X)		
Pelaez-Rodriguez et al.^31^	Mondonedo (Spain)	T, H, WindD, windV, Pressure	Vis	LSTM, CNN, GRU, RNN
Yu et al.^1^	Nanjing (China)	PM2.5, H	Vis	MPR, Correlation
		Vis(−) and OC(+), Vis(−) NH_4_(+)		
		Na, Mg, NH_4_, Ca, K, F, NO_3_		
Zhao et al.^29^	Shenyang	Vis—Wind (R = 0.60), Vis—RH (R = 0.50)	Vis	Correlation
		Vis—T (R = 0.15), Vis—PM2.5 (R = 0.51)		
		Vis—PM1 (R = 0.50)		
Tandon et al.^55^	Dehli, semirad (India)	PM2.5(−), DP(+), T(+), windV(+)	Vis	Non—linear decomposition
Deng et al.^27^	Pearl River Delta (China)	PM1(−), PM2.5(−), PM10(−)	Vis	MPR
Ma et al.^18^	Hangzhou (China)	Vis—OC(R = − 0.492)/EC(R− 0.443)/TC(R = − 0.531)	Vis	Correlation
		Vis—NH_4_(R = − 0.783)/SO_4_(R = 0.678)/F(R = − 0.561)/Cl(R = − 0.531)/NO3(R = − 0.782)		
Lee et al.^26^	5 City: Korea (Seoul, Daegu, Busan, Ulsan, Ullugelo)	Vis—PM1(R = − 0.15 to − 0.43)/SO_2_(R = − 0.04 to − 0.25)	Vis	Correlation
		Vis—SO_2_(R = − 0.04 to − 0.25)/O3(R = − 0.03 to − 0.18)		
		Vis—NO_2_(R = − 0.06 to − 0.33)/CO(R = − 0.05 to − 0.29)		

T, temperature; RH, relative humidity; DP, deplection point; Vis, visibility; Press, pressure; WindD, direction of wind; WindV, velocity of wind; OC, organic carbon; EC, elemental carbon; TC, total carbon; NDVI, normalized difference vegetation index; Elev, elevation; lU, distance to nearest industrial region; AOD, aerosol optical depth; Traffic, vehicle traffic volume; t, time; x; y, spatial data; GRU, gated recurrent unit; RNN, recurrent neural network; LSTM, long short term memory; MPR, exponential function; RF, random forest; SVM, support vector machines; MLP, multilayer perceptron.

Air quality and Vis issues in Poland remain relatively underexplored in scientific research, despite the country experiencing some of the lowest air quality levels in Europe^32–34^. Poland is notably exceptional in terms of both emissions and the concentration of PM as well as gaseous precursors of PM^4,35^. The concentration of PM in the ambient air across Poland is predominantly influenced by transportation and energy production. Notably, the energy sector has been identified as a major contributor to elevated PM concentrations in Polish regions compared to other developed European countries. This sector, relying heavily on hard and brown coal, not only acts as a source of PM but also releases gaseous precursors, particularly organic compounds, sulfur and nitrogen oxides, and ammonia. So-called communal emissions have become a significant concern in Poland, particularly in suburban and city centres in southern Poland. This issue is closely linked to coal combustion in small, low-efficiency household furnaces, contributing to smog episodes in winter^36,37^. Simultaneously, it leads to consistently high PM concentrations in certain areas throughout the year. Acknowledging the boundary-less nature of air, it becomes evident that while these issues may have localised origins, they also have continental implications. The impact of air quality in Poland extends beyond national borders and is observable on a European scale^38–40^. Therefore, monitoring air quality, including Vis, becomes of paramount importance, as it serves not only to protect the health and well-being of citizens but also to address transboundary air pollution challenges.

Current Vis forecasting models typically account for meteorological conditions and selected contaminant concentrations (e.g. PM10, PM2.5, SO_2_, NO_2_, O_3_, etc.), with limited consideration for the chemical composition of PM^41,42^. Furthermore, due to constraints in existing calculation methods, accommodating variables with a non-linear influence on Vis has proven challenging. Typically, a dependency is observed between PM concentration and the relative air H, representing a substantial simplification. Machine learning methods, when applied to predict Vis, often neglect the impact of selected input data on simulation results, a critical aspect for comprehending atmospheric mechanisms.

In response to these limitations, we propose a novel approach to Vis modeling, incorporating PM1 concentration, chemical composition, and meteorological conditions. Our main objective is to develop an effective tool for forecasting Vis that considers the complex interactions between atmospheric factors, air quality, and the chemical composition of PM1. The devised methodology encompasses: (i) modeling Vis using machine learning, (ii) identifying data clusters through unsupervised learning (k-means), and (iii) conducting individual global sensitivity analysis (GSA) for each cluster. The multi-layer perceptron (MLP) method is employed to predict Vis. The integration of k-means clustering and GSA facilitates establishing relationships among meteorological conditions, air quality, elemental composition, and Vis, accounting for the intricate interaction between input data. The ultimate objective is to comprehend more precise mechanisms influencing Vis in the context of atmospheric conditions. This can contribute to a more efficient monitoring and management of air quality, especially in the context of Polish conditions where these issues are particularly crucial.

## Results

### Mass concentrations of PM1

**Figure 1:**
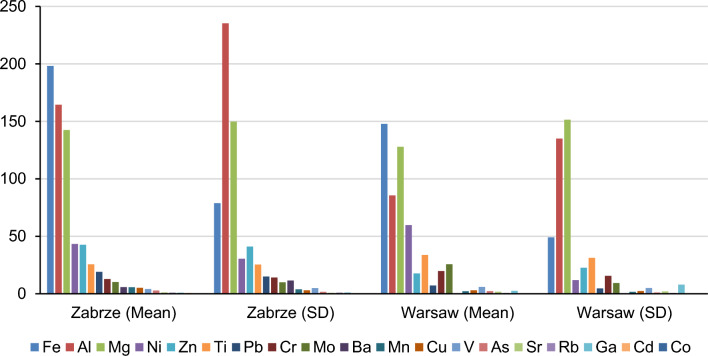
Statistical summary of elemental compositions (ng/m^3^) at the sampling station.

During the PM1 concentration measurement campaign, 120 samples were successfully collected in each of the two cities analyzed. In Zabrze, the 24 h PM1 concentrations ranged from 6.5 to 198.5 µg/m^3^ (± 30.1 µg/m^3^), while in Warsaw, they ranged from 4.7 to 39.0 µg/m^3^ (± 7.1 µg/m^3^)^43–47^. Regarding the composition of PM1, it’s notable that the collective mass of all elements represents a minor fraction of PM1. In Zabrze, this fraction accounted for approximately 2.5% of PM1 overall (around 6.1% in summer and approximately 1.8% in winter), while in Warsaw, it constituted 4.7% of PM1 (approximately 5.83% in summer and 3.7% in winter). Figure 1 illustrates the average concentration of elements in PM1, along with their standard deviations over the measurement period. In Warsaw, dominant components of PM1 included Fe, Al, and Mo, which collectively represented about 66% of the measured metal concentrations. Conversely, in Zabrze, the primary components were Fe, Al, and Mg, accounting for approximately 74% of the total heavy metal concentrations observed^48,49^.

### Selection of input data for the model

In the context of selecting input data for the Vis prediction model, a thorough analysis of the correlations between pairs of meteorological conditions and elements was conducted. This analysis aimed to identify significant relationships and determine which variables should be included or excluded from the model. Specifically, a correlation coefficient (R_ij_) exceeding 0.89 indicated a strong correlation, prompting consideration for the removal of one of the variables to avoid multicollinearity.

**Figure 2:**
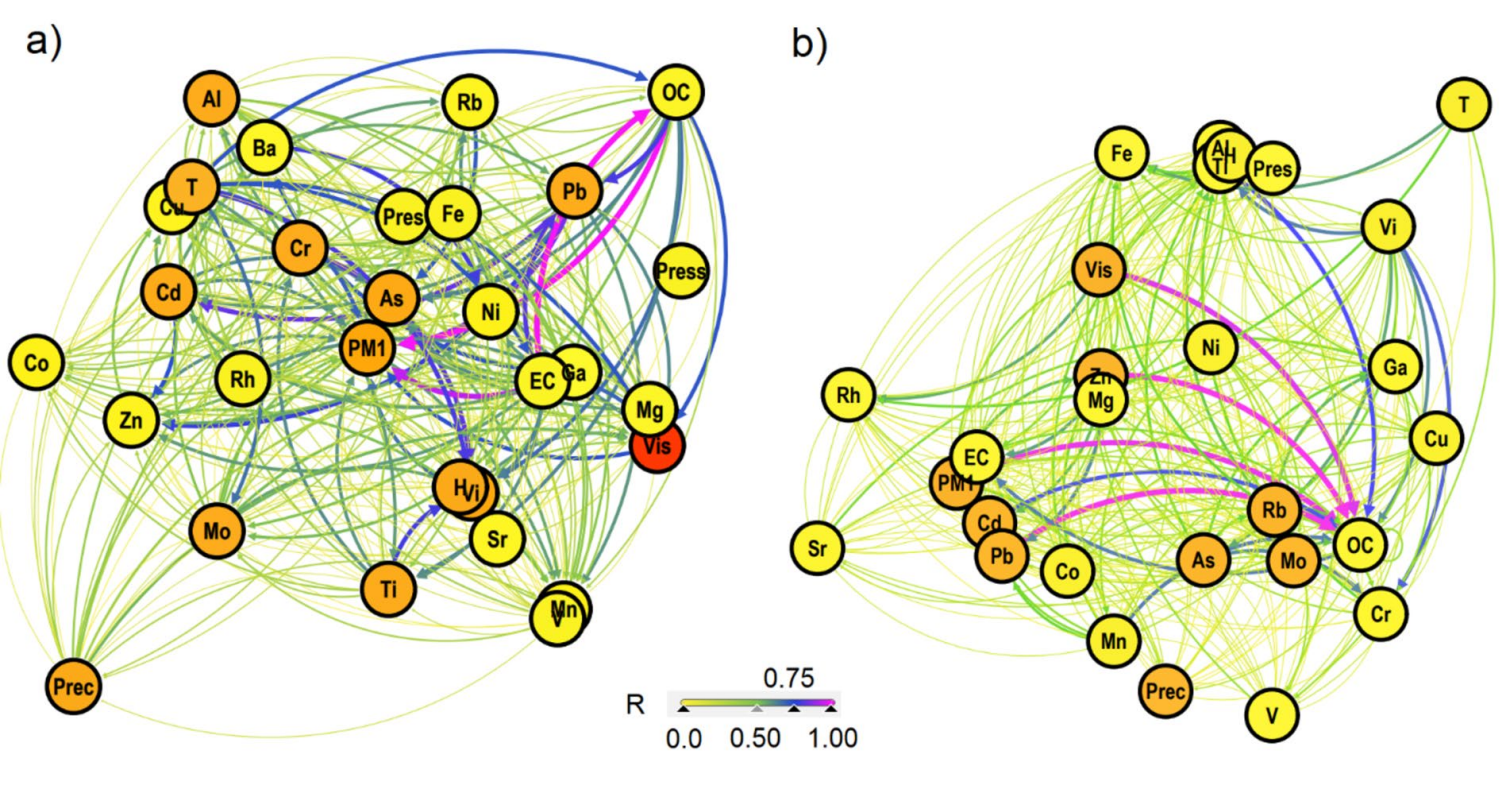
Network analysis of the correlations between variables describing meteorological conditions, PM chemical composition, and visibility for (**a**) Zabrze and (**b**) Warsaw.

Strong correlations (R_ij_ > 0.60) were observed between Vis and temperature (T) (R_ij_ = 0.71), as well as RH (R_ij_ = 0.70) in Warsaw (Fig. 2b), while in Zabrze, T exhibited the most significant impact on Vis, along with organic carbon (OC) and elemental carbon (EC) (R_ij_ = 0.68, 0.67, and 0.65, respectively) (Fig. 2a). For Warsaw, the average correlation of (R_ij_ = 0.40–0.60) between Vis and OC (R_ij_ = 0.50) and EC (R_ij_ = 0.52) was observed.

Furthermore, both cities demonstrated a strong correlation between Vis and PM1 concentrations (R_ij_ = 0.50–0.69). Noteworthy correlations (R_ij_ ≥ 0.40) were found between Vis and specific elements, such as Pb, V, Mo for Zabrze, whereas in the case of Warsaw—for Zn, Pb, Ti and Mo.

For Warsaw, correlations of R_ij_ ≥ 0.89 were noted between OC–PM1 and OC–EC, and in the case of Zabrze—between OC–EC, PM1–OC and EC–PM1. For Warsaw, it has been shown R_ij_ ≥ 0.7 for Vi–Cr, Vi–Ti, Pb–Zn, Cr–As, and between OC, EC and heavy metals: Zn, Pb. It has been demonstrated for Zabrze R_ij_ ≥ 0.7 for Vi–Ti, Vi–T, Zn–Cd, Cd–Pb, Ba–Ni, Pb–Zn, Pb–Cd, and between Pb i OC, EC.

In an aim to complete the above analyses and adequately select input data for the model, Fischer–Snedecor calculations were additionally carried out (Table S1). The orange colour in Fig. 2 marks variables for which *p* < 0.05 was obtained and which were ultimately accounted for in the model. These results confirmed the occurrence of multicollinearity, due to which independent variables for which R_ij_ ≥ 0.9 were removed from father analyses.

Keeping the above products and results Fischer–Snedecor tests (Table S1) in mind, the following input data: PM1, T, RH, precipitation (Prec) along with elements such as Pb, V, Mo, As, Cd, Ti, Al. and Cr were assumed for predicting Vis in Warsaw. In the case of Zabrze, identical meteorological conditions were chosen, with the addition of PM; the elements Pb, V, Mo, As, Cd, Ti, Al, and Cr were adopted for the model. The adopted input data for the Warsaw and Zabrze models were necessary due to the assumed research topic, i.e., determining the influence of elements on Vis, a topic on which very few studies were limited to a single measurement point. The selected combination of input data was aimed at identifying the influence of heavy metals on air Vis, which has been poorly recognized to date. The calculations revealed strong correlations between key air quality parameters (OC, EC) and pollutants (PM1), leading to the elimination of these variables from the developed model for Warsaw and Zabrze (Table S7). Ultimately, the study considered a variant that includes input data for the MLP model (Warsaw, Zabrze) according to the Fisher–Snedecor method. However, it also considered a limited scope (meteorological conditions, air quality, parameters describing air quality) and a full range of data—see “Selection of input data for the model” section.

### Cluster analysis

**Figure 3:**
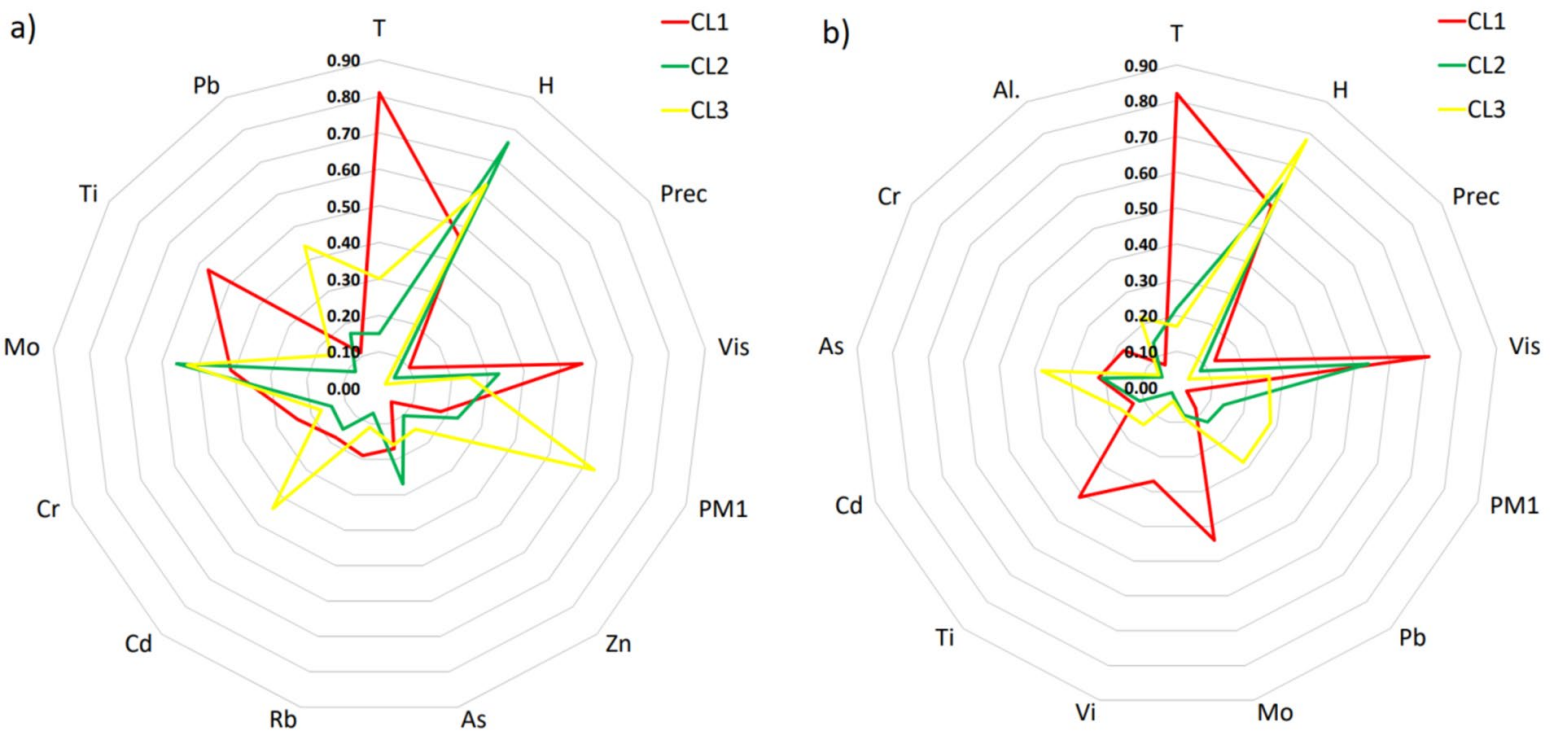
Normalized average values of variables for CL1, CL2, and CL3 clusters for (**a**) Warsaw, and (**b**) Zabrze.

Hierarchical Cluster Analysis categorized measurement data, including meteorological conditions, air quality, elemental composition, and Vis, into three distinct clusters for both Warsaw and Zabrze (Figs. S1–S4). In Warsaw, Cluster CL1 exhibited the highest visibility (Vis = 11.44 km), while Cluster CL3 had the lowest (Vis = 1.36 km) (Fig. 3a). Cluster CL1 also showed significantly higher values for Ti, Cr, As, and Rb compared to CL3, with respective ratios of 3.73, 1.35, 1.38, and 1.76 (Table S3). Conversely, PM1, Zn, Pb, Mo, and Cd values were lowest in CL1, with ratios ranging from 0.23 to 0.66 times those of CL3 (Fig. 3a).

In Zabrze, Cluster CL1 had the highest visibility (Vis = 9.58 km), while CL3 had the lowest (Vis = 4.71 km) (Fig. 3b). Cluster CL1 exhibited significantly higher values for Mo, V, Ti, and Cr compared to CL3, with ratios of 4.73, 7.25, 2.88, and 2.78, respectively (Table S4). Conversely, PM1, Pb, Cd, As, and Al values were lowest in CL1, with ratios ranging from 0.41 to 0.8 times those of CL3. Higher values of T and Prec were consistently observed in CL1 compared to CL3 for both Zabrze and Warsaw (Fig. 3).

### Multi-layer perceptron model (Warsaw, Zabrze)

**Figure 4:**
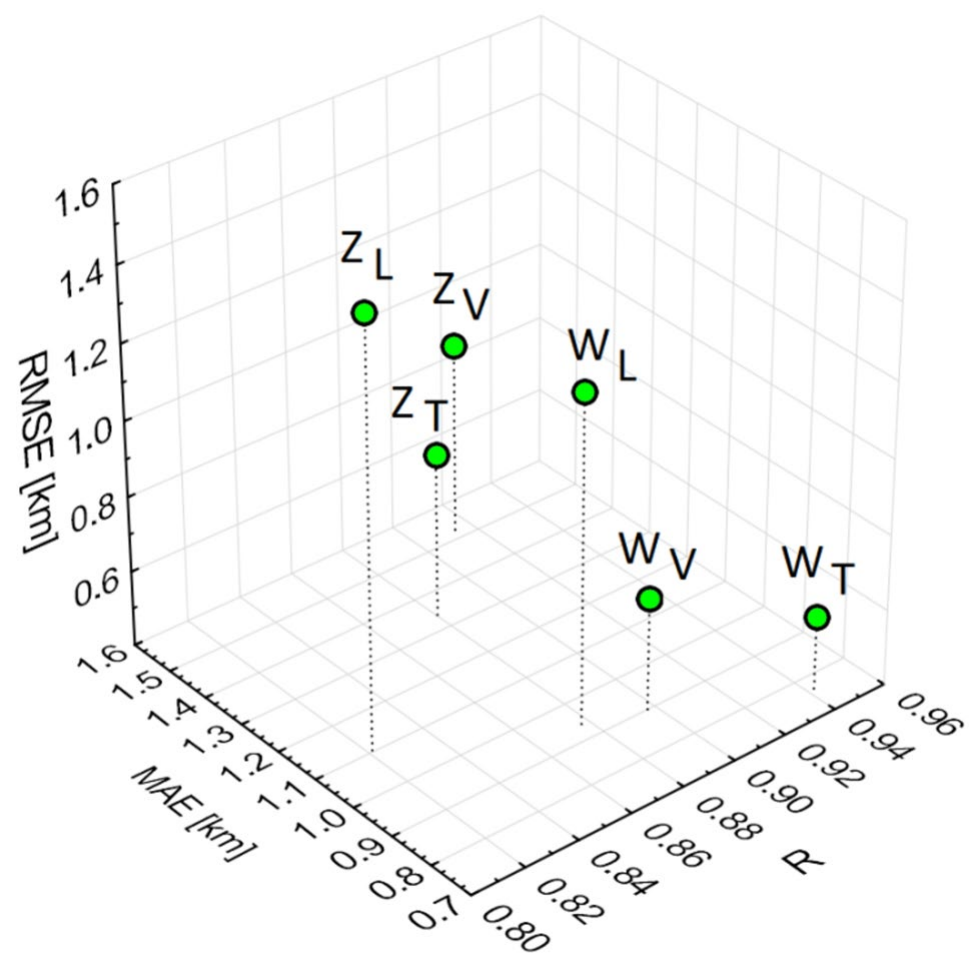
Relationship RMSE = f(MAE, R) for the learning set (L), test set (T), and validation set (V) for Warsaw (W) and Zabrze (Z).

In Warsaw, the study showed that the variant including the full set of input data (meteorological conditions, air quality, meteorological parameters, and chemical composition) achieved the best alignment of forecasts with measurements (R = 0.95, MAE = 0.80, RMSE = 0.83 for the test set) (Fig. 4). Variants omitting certain input data based on the Fisher–Snedecor test showed greater forecast errors, with R values decreasing to 0.91 and 0.83, and higher MAE and RMSE values accordingly (Table 2).

**Table 2:** Evaluation of MLP models with various input variants.

Variant	R	MAE	RMSE	R	MAE	RMSE	R	MAE	RMSE
	Learning			Test			Validation		
Zabrze									
(a)	0.78	0.99	1.15	0.80	0.99	1.15	0.80	0.92	1.14
(b)	0.92	0.87	0.96	0.96	0.82	0.94	0.95	0.82	0.96
(c)	0.85	0.93	1.00	0.84	0.90	1.02	0.86	0.87	1.03
Warsaw									
(a)	0.82	1.21	1.73	0.83	1.20	1.72	0.83	1.22	1.70
(b)	0.95	0.80	0.83	0.96	0.79	0.85	0.95	0.83	0.85
(c)	0.90	0.85	0.90	0.91	0.87	1.37	0.90	0.92	0.93

In Zabrze, the smallest forecast errors for Vis were obtained when using the full variant (R = 0.96, MAE = 0.82, RMSE = 0.94) (Table 2). The largest errors occurred when the chemical composition was omitted (R = 0.80, MAE = 0.99, RMSE = 1.15), with intermediate errors observed for variants based on Fisher–Snedecor test results (R = 0.84, MAE = 0.90, RMSE = 1.02).

### Global sensitivity analysis

#### Identification of theoretical distributions for models (Warsaw, Zabrze)

The analyses performed (Tables S5, S6) have demonstrated that empirical distributions accurately model the theoretical distributions of variables in both Warsaw and Zabrze. This conclusion is supported by rigorous statistical tests, including Kolmogorov–Smirnov, Chi-Square, and Anderson–Darling tests. For Warsaw, the variables PM1, Zn, As, Rb, Cd, Pb, Cr, T, and RH exhibit a close fit to the Generalized Extreme Value distribution, indicating their extreme value behavior under specific conditions. Variables Vi and Prec adhere well to Johnson’s distribution, which characterizes their complex distribution patterns. The variable Ti shows a mixed distribution, reflecting its diverse influences and variations.

Similarly, in Zabrze, the analysis reveals that PM1, Vi, As, Cd, Pb, Cr, Mo, and RH are appropriately modeled by the Generalized Extreme Value distributions, highlighting their extreme characteristics in the local environmental context. Al and Ti align well with Johnson’s distribution, reflecting their distinct probabilistic structures. Meanwhile, the variables T and Prec display characteristics best captured by the mixed distribution, underscoring their variability influenced by multiple factors.

#### Meteorological conditions and PM1

**Figure 5:**
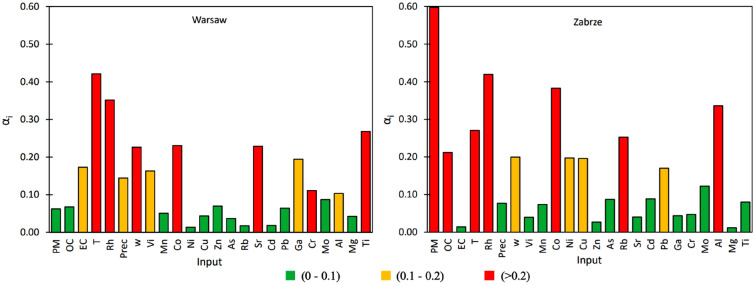
Sensitivity coefficient (αi) for C1, C2 and C3 variables: (**a**) Warsaw, (**b**) Zabrze.

Within the analyzed atmospheric conditions, the most significant factors influencing visibility in Zabrze were temperature (T) (αi = 0.421), relative humidity (RH) (αi = 0.352), and wind speed (w) (αi = 0.226), whereas PM1 exhibited only αi = 0.063 (Fig. 5). Regarding air quality parameters, EC had a crucial impact on visibility in Zabrze (αi = 0.173), while OC showed merely αi = 0.063. The lower influence of PM1 on visibility compared to EC can be explained by the high correlation coefficients (R_ij_ > 0.9) between PM1 and OC, and OC and EC. In Warsaw, similar to Zabrze, among the meteorological conditions, relative humidity (RH) (αi = 0.420), temperature (T) (αi = 0.271), and wind speed (w) (αi = 0.200) significantly influenced visibility, with PM1 (αi = 0.597) having a key impact on visibility (Fig. 5). In terms of air quality parameters, the most substantial influence on visibility in Warsaw was from organic carbon (OC) (αi = 0.212). The relatively minor impact of EC on visibility compared to OC and PM1 can be explained by the high correlation between EC and OC (R_ij_ = 0.81) and between PM1 and OC (R_ij_ = 0.90).

#### Elements

Through GSA, several factors were identified for their significant impact on visibility in both Zabrze and Warsaw (Fig. 5). In Zabrze, the analysis highlighted that Ti (αi = 0.268), Co (αi = 0.231), Sr (αi = 0.228), Ga (αi = 0.114), and Vi (αi = 0.163) played crucial roles in influencing visibility conditions. Each of these elements contributed distinctively to the variability observed in visibility measurements. Conversely, in Warsaw, different factors emerged as key influencers of visibility: Co (αi = 0.383), Rb (αi = 0.253), Ni (αi = 0.197), Cu (αi = 0.196), and Pb (αi = 0.170). These elements exhibited varying degrees of influence, contributing significantly to the observed variations in visibility levels across different conditions and locations.

## Discussion

### Elemental composition and concentration variability of PM1

In our study, PM1 concentration varied significantly, ranging from 6.5 to 198.5 µg/m^3^ and from 4.7 to 39.0 µg/m^3^ for Zabrze and Warsaw, respectively. PM1 levels in Poland were higher than levels measured at the background station in Finland (4.3–3.8 µg/m^3^), similar to those at an urban station in Italy (22 ± 6 µg/m^3^) and an urban station in Turkey (22.1 ± 6.4 µg/m^3^), but lower than in Hong Kong Kong (40.4 ± 19.4 µg/m^3^) or an urban background stations in China (127.3 ± 62.1 µg/m^3^)^43–47^. Despite the varying concentrations, the contribution of elemental content to PM1 was relatively minor. In Zabrze, elements constituted approximately 2.5% of PM1, with seasonal variations showing about 6.1% in summer and 1.8% in winter. In Warsaw, the elemental contribution was around 4.7% of PM1, with 5.83% in summer and 3.7% in winter. These shares are higher compared to those reported in other cities, such as Toronto (1.2%)^48^ and Milan (1.4%)^49^. This suggests that while PM1 levels are influenced by local and regional factors, the elemental composition remains a small but notable fraction of the total particulate matter.

Our findings reveal that Fe, Al, and Mo were the predominant components of PM1 in Warsaw, collectively constituting approximately 66% of the metal concentrations during the measurement period. This dominance suggests significant industrial and vehicular emissions as potential sources, considering the urban and industrial landscape of Warsaw^50,51^. Similarly, Fe, Al, and Mg were the predominant elements in Zabrze, comprising about 74% of the heavy metal concentrations. This higher proportion underscores the influence of industrial activities and possibly coal combustion, reflecting the region’s industrial profile^52,53^. The disparity in the elemental composition between the two cities highlights the varying sources and environmental impacts influencing PM1 levels in urban and industrial areas.

### Application of machine learning model for simulating Vis

Literature data has substantiated the application of machine learning methods (SVM, MLP) in predicting heavy metal concentrations in PM1 based on air quality (SO_2_, NO_2_, CO, O_3_, PM2.5) and meteorological conditions (T, RH, pressure, windS)^28^. The resulting correlation values (R > 0.85) suggest that machine learning methods serve as valuable tools for simulating intricate processes that describe the chemical mechanism of air composition changes. This affirmation is reinforced by Pelaez-Rodriguez et al., who used LSTM, GRU and CNN models to predict Vis based on T, RH, windD, windS and pressure obtaining successful results^31^.

In alignment with previous research, Vis analyses traditionally considered standard meteorological conditions (T, RH, windD, windS, pressure) air quality (NO_x_, PM, O_3_, CO etc.), and elemental composition (Na^+^, NH^4+^, F^−^, Mg^2+^, etc.)^29,31,54,55^. However, recent studies have demonstrated that heavy metals can also impact Vis. Up to now, a few studies have addressed impact of individual metals in PM (e.g. PM10 and PM2.5) on vis, but they only connected these findings with single parameters such as meteorological conditions or air quality^3,56,57^. Therefore, in the present work, a non-standard set of input data was adopted, which allowed for finding the relationships between Vis and meteorological conditions (T, RH, Prec), air quality (PM1), and heavy metal contents, which had not been analyzed in such a wide scope. This broadened scope of analysis is crucial, as it explores uncharted relationships not previously scrutinized comprehensively. Recognizing the interpretative challenges in machine learning models, a GSA model was recommended, enhancing the understanding of relationships and interactions between input data and air Vis—a novel approach not commonly employed in Vis-related calculations^11,28,31^.

The modification of the Genetic Algorithm (GSA) with the integration of k-means clustering has illustrated promising capabilities in delineating interconnected mechanisms governing Vis. These mechanisms are intricately linked to meteorological conditions, air quality parameters, and the presence of heavy metals.

The outcomes derived from the augmented GSA model resonate with the simplified analyses undertaken by Yu et al.^1^, and Zhao et al.^58^, shedding light on the multifaceted relationships between Vis and PM concentrations (specifically PM1/PM2.5) across varying ranges of RH variability. Notably, the approach adopted in our study transcends the scope of previous analyses conducted by Yu et al.^4^ and Zhao et al.^3^, facilitating a deeper exploration of the nuanced mechanisms operating in multidimensional contexts. Additionally, we draw comparisons with the methodology proposed by Li et al.^46^.The authors utilized a hybrid machine learning model to predict Vis and reported an R of 0.71 in their cross-validation. In contrast, our augmented GSA model demonstrates superior predictive performance, with R values exceeding 0.86 in various scenarios. Compared to these traditional approaches, the augmented GSA model offers a comprehensive analysis encompassing a wider array of input variables, including meteorological conditions, air quality parameters, and heavy metal contents. This holistic approach enables a more accurate prediction of Vis, as showcased by the enhanced predictive capabilities demonstrated in our study.

### Influence of input data (meteorological conditions, air quality, element composition) on Vis

This study demonstrates a positive correlation between T and Vis, influenced by RH, Prec and elemental concentrations. This observation aligns with findings from previous research conducted by Yu et al. for Nanjing^1^, Zhao et al. for Shenyang (2010–2012 period)^58^, and Tandon et al.^55^, who developed a model of time series for Delhi, considering a cyclic and acyclic trend. Deng et al.^59^, analysing data from five cities in China (Xiamen, Fuzhou, Taipei, Taichung, Tainen) during 1973–2001, similarly reported a positive correlation between Vis and air T^27^. The authors highlighted the influence of local conditions on this relationship, resulting in variable correlation coefficients (R = 0.51–0.77).

GSA conducted for Warsaw and Zabrze confirmed that an increase in RH leads to a decrease of Vis, consistent with trends observed by Yu et al.^1^ and Zhao et al.^58^. Majewski et al.^60^, based on a 7-year series of Vis observations, meteorological conditions, and air quality, confirmed the relationships revealed in the present work. Tandon et al.^55^, in the described model covering the decomposition for two cycles, showed that PM2.5, DP (dew point), and windS influence the cyclic trend, whereas the acyclic trend is influenced by DP, windS, and PBL (planetary boundary layer) respectively. Pelaez-Rodriguez et al.^31^, in machine learning models (RNN, CNN, GRU, LSTM), also accounted for RH and PM1 for Vis forecasting. However, the absence of GSA in their work precludes a detailed examination of the influence of the individual input data without further analysis.

## Conclusion

The proposed methodology for predicting Vis, incorporating PM1 concentrations, chemical composition, and meteorological conditions, exhibits remarkable adaptability and can be reliably applied to analogous geographical regions. The unique characteristics of the two study areas in Poland, Zabrze and Warsaw, each facing distinct meteorological conditions and emission sources, underscore the universality of the proposed approach. Zabrze, situated in the southern region, is predominantly influenced by municipal and industrial emissions, whereas Warsaw in central Poland experiences a substantial impact from road transport emissions, as extensively documented in previous authors research.

The study underscores the effectiveness of the MLP model in predicting Vis. Furthermore, the synergistic application of the k-means clustering method and GSA proves invaluable in deciphering intricate mechanisms governing changes in chemical compounds within PM and air, subsequently influencing Vis. Notably, the research emphasises the substantial impact of meteorological conditions and air quality on the nuanced relationships between PM chemical composition and Vis. This revelation is consistently supported by calculations conducted for both Warsaw and Zabrze, providing nuanced insights into the interplay of various factors influencing Vis in these regions.

## Methods

The two identical PM1 sampling sets were deployed in Zabrze (south of Poland, λE = 18° 46′; φN = 50° 18′) and Warsaw (central Poland, λE = 21° 02′; φN = 52° 09′). Each set included a low-volume sampler (2.3 m^3^/h, Twin PM; Zambelli Milan, Italy) coupled with a PM1 sampling head (TSI; MN, USA); utilising quartz fiber filters (Whatman, Ø47 cm, UK). Sampling occurred during two distinct periods: June to August 2014 (spanning 60 days in summer, a non-heating season) and January to March 2015 (spanning 60 days in winter, a heating season), simultaneously in both locations.

The chemical composition of PM varied significantly between the two cities^11,36,61^. The Lab OC-EC Aerosol Analyzer (Sunset Laboratories Inc., USA) was employed to determine the OC and EC content of PM1, following the EUSAAR protocol. Elemental composition (Vi, Mn, Co, Ni, Cu, Zn, As, Rb, Sr, Cd, Pb, Ga, Cr, Mo, Al, Mg, Ti) was assessed using High-Resolution Inductive Coupled Plasma-Mass Spectrometry (HR-ICP-MS, 6100 DRC-e Perkin Elmer, Waltham, MA, USA). Iron content was determined through Inductively Coupled Plasma Optical Emission Spectroscopy (ICP-OES; Avio 200, USA). The methods were validated against Certified Reference Material to ensure accuracy and reliability (recoveries ranged from 92 to 109%). The analysis was performed on samples collected over a 24-h interval. Detailed information regarding the chemical analysis of PM and sample preparation can be found in the previous papers^11,62,63^.

Various meteorological parameters, including T, solar radiation intensity (Rad), RH, Prec, atmospheric pressure, windS and Vis (ranging from 10 to 50 km) were measured. The Vis measurements were carried out using a Vis meter equipped with an atmospheric phenomenon detector (Vaisala FS11, wavelength 875 nm). Air T and RH were measured using a Vaisala HMP 155 sensor with accuracy for T ranging from − 80 °C to + 20 °C at ± (0.226–0.0028 × temp.)°C and from + 20 °C to + 60 °C at ± (0.055 + 0.0057 × temp.)°C, and for RH ranging from + 15 °C to + 25 °C, the accuracy is ± 1% for 0–90% RH and ± 1.7% for 90–100% RH. Rad was measured with two sensors. The Kipp & Zonen CSD 3-M3 sunlight sensor, which has a spectral range of 400–1100 nm and an accuracy of over 90% per month for sunlight hours, and the Kipp & Zonnen CMP11 pyranometer with a spectrum range of 310–2800 nm. The CMP11’s sensitivity is 7 to 14 µV/W/m^2^. The Teodor FRIDRISCH rain gauge type 7051.1000 is used to measure precipitation, with a collecting surface of 200 cm^2^ and a measurement sensitivity of 0.1 mm. For atmospheric pressure, the Vaisala PMT16A sensor is employed, featuring an accuracy of ± 0.3 hPA. WindS were measured using the VAISALA WS425-B2A1B sensor, with an accuracy is at 0.135 m/s. The meteorological stations were colocated with other monitoring equipment in both Zabrze and Warsaw. Hourly results for meteorological parameters were averaged over 24-h intervals from 12:00 to 12:00 h; aligning with the timing of diurnal PM1 samples. The temporal resolution of the data was consistent across all measurements, providing a comprehensive overview of the atmospheric conditions during the study period.

### Statistical analyses

Before embarking on the construction of calculation models, the dataset underwent preliminary analyses. Initially, Spearman’s correlation was indicated between variables. To expand the scope of the analyses, cluster analysis was carried out to analyse the relationships between air Vis, meteorological conditions, and elemental composition. The subsequent phase of analysis involved the identification of dependent variables through the Fischer–Snedecor test, specifically for the Vis prediction model utilising artificial neural networks in both Zabrze and Warsaw. Recognising the inherent challenges in interpreting the impact of input data on machine learning model outcomes, a GSA was undertaken. This GSA utilised the polynomial regression method with interactions to comprehensively understand the influence of variables on the results of simulations.

#### Selection of input data for the model

In this study, three variants of input data were considered for model development:

- air quality parameters (OC, EC), meteorological conditions (w, T, RH), and air pollutants (PM1),
- data from variant (a) along with chemical composition including heavy metals (Mn, Co, Ni, Cu, Zn, As, Rb, Sr, Cd, Pb, Ga, Cr, Mo, Al, Mg, Ti),
- data selected based on the Fisher–Snedecor test values, a commonly used practice in the initial stages of machine learning model development^64,65^. The process of selecting input data in the present work relied on the outcomes of both correlation analysis and the Fischer–Snedecor test. To visually represent the correlation results among input data, the Gephi 6.0 program was applied. However, to select the independent variables for the developed machine learning model, intended for simulating Vis, the Spearman’s correlation of independent variables was rigorously evaluated.

In the subsequent phase, the Fischer–Snedecor test was applied to calculate the *p* value at the predetermined significance level. Variables with a calculated *p* value meeting or falling below *p* ≤ 0.05, considered for inclusion in the model development.

For the final analyses, the model with the best alignment between forecast results and measurements was selected, using the fit metrics discussed in the “Multilayer perceptron” section.

#### Cluster analysis

The study employed cluster analysis, starting with hierarchical clustering as the primary technique. Initially, each data point was treated as an individual cluster, which was then progressively merged based on their similarity until forming cohesive groups. To assess the similarity between clusters and determine merging distances, established criteria were applied. The analysis utilized the Euclidean distance function along with Ward’s method, which incorporates variance analysis to measure distances between clusters. This approach facilitated the creation of meaningful clusters based on the similarity of data points^66,67^. Subsequently, the k-means method, also known as the centroid algorithm, was applied. This method involved determining the optimal number of clusters (k) using the results from the hierarchical cluster analysis. Once the clusters were identified, the k-means method was utilized to characterize the variability ranges within each cluster for variables such as Vis, influenced by meteorological conditions and the elemental composition of PM. The theoretical foundations of the method are thoroughly elucidated in the study by Bayo and Lopez-Casellanos^68^.

#### Multilayer perceptron

Artificial neural networks are widely utilized in machine learning applications for modeling various phenomena such as air quality, visibility, and meteorological conditions^69,70^. Their effectiveness is underscored by their successful application in MLP-type networks (Fig. S1), where input signals (x_k_) undergo multiplication by the values of weighs (w_ik_) and are subsequently transmitted to neurons of the hidden layer. The summation of individual neurons in this layer follows the relationship:

1 $$z_{i} = \mathop \sum \limits_{k}^{m} x_{k} \cdot w_{ik} - b_{i}$$

where m and n refer to the number of neurons in the preceding and current layer of the network, whereas b_i_ refers to the threshold (so-called bias). The obtained totals (z_i_) are subjected to transformation using the linear or non-linear activation function (f), and are carried over to the output neurons. The optimisation of weight values (w_ik_) for individual neurons during the network’s learning process.

Due to the limited amount of data available at the model training stage, hyperparameter identification for the MLP models was performed using fivefold cross-validation to reduce the uncertainty of the simulation results. A 60/20/20 split of the dataset, where 60% of the data was used for training, 20% for validation, and 20% for testing, was incorporated. This method allowed us to effectively train and evaluate our model, ensuring that it is robust and generalizes well to new data. This approach ensures the development of models with generalization capabilities, based on measurement data collected from 50^72^ or even 20^71^ experiments.

A comprehensive description of MLP model calibration procedure and goodness-of-fit analysis is given in Sect. S1.

#### Global sensitivity analysis

To gauge the impact of input data on the results of Vis simulations using the MLP method, a GSA was employed based on analytical dependencies represented by second-degree multivariate polynomial regression. When dealing with strongly non-linear processes, the application of MRP models can be limited, leading to potentially unreliable GSA results.

The assumed calculation procedure encompasses the following steps:

- indicating the theoretical distributions of variables—Sect. S1,
- Monte Carlo simulation (Imana-Conovera) accounting for the correlation of data—Sect. S2,
- estimation of multivariate polynomial regression model parameters (for k clusters) in the form of:
  2 $$Vis^{*} = \alpha_{0} + \mathop \sum \limits_{i = 1}^{j} \alpha_{i} \cdot x_{i}^{*} + \mathop \sum \limits_{i < j}^{n} \mathop \sum \limits_{j = 2}^{n} \alpha_{ij} \cdot x_{i}^{*} \cdot x_{j}^{*}$$

where α_0_, α_1_, α_2_, α_j_—coefficients estimated by the method of least squares; x_j_*, Vis*(k)—normalised input/output variables calculated in accordance with Sect. S2.

### Supplementary Information

Supplementary Information.
