# Supplementary material for: Machine learning analysis of PM1 impact on visibility with comprehensive sensitivity evaluation of concentration, composition, and meteorological factors
Source: Sci Rep. 2024 Jul 20;14:16732. doi: 10.1038/s41598-024-67576-8 (PMC11271544; doi:10.1038/s41598-024-67576-8)
Supplement: Supplementary file 1 — Supplementary Information. [file 41598_2024_67576_MOESM1_ESM.docx]

**Machine Learning Analysis of PM1 Impact on Visibility with Comprehensive Sensitivity Evaluation of Concentration, Composition, and Meteorological Factors**

**Grzegorz Majewski**1,***, Bartosz Szelag**1**, Wioletta Rogula-Kozłowska**2**, Patrycja Rogula-Kopiec**3**, Andrzej Brandyk**1**, Justyna Rybak**4**, Maja Radziemska**1**, Ernesta Liniauskiene**5**, and Barbara Klik**1

1Institute of Environmental Engineering, Warsaw University of Life Sciences, Warsaw, 02-776, Poland

2Fire University, Faculty of Fire Safety Engineering, Warsaw, 01-629, Poland

3Institute of Environmental Engineering, Polish Academy of Sciences, Zabrze, 41-819, Poland

4Faculty of Environmental Engineering, Wrocław University of Science and Technology, Wrocław, 50-370, Poland 5Department of Hydrotechnical Engineering, Faculty Environmental Engineering, Kaunas Forestry and Environmental Engineering University of Applied Sciences, Girionys, Kaunas, LT-53101, Lithuania

*grzegorz_majewski@sggw.edu.pl

**Support Information**

**Section S1**

Data for the construction of the model were divided into three sets: learning (70%), test (15%) and validation (15%). To indicate weights (w_ik_), the Broyden – Fletcher – Goldfarb –Schano algorithm was used. The structure of the MLP models for Warsaw and Zabrze was determined using 10-fold cross-validation. The number of neurons was assumed in the M ÷ 2·M+1 scope, where: M – the number of input data. The STATISTICA 13.0 program was used to construct the model. To assess the predictive abilities of the models, the following were assumed:

- determination coefficient (R^2^):

$R=\frac{\sum_{i=1}^{N} \left( y_{,pom}-\bar{y_{i,pom}} \right)\cdot\left( y_{,obl}-\bar{y_{i,obl}} \right)}{\sqrt{{\sum_{i=1}^{N} \left( y_{,pom}-\bar{y_{i,pom}} \right)}^{2}}\cdot\sqrt{{\sum_{i=1}^{N} \left( y_{,obl}-\bar{y_{i,obl}} \right)}^{2}}}$ (1S)

- mean absolute error (MAE):

$MAE=\frac{1}{N}\cdot\sum_{i=1}^{N} \left| y_{i,pom}-y_{i,obl} \right|=\frac{1}{N}\cdot\sum_{i=1}^{N} e$ (2S)

- root mean square error (RMSE):

$RMSE=\sqrt{\frac{1}{N}\cdot\sum_{i=1}^{N} \left( y_{i,pom}-y_{i,obl} \right)^{2}}$ (3S)

where: y_pom_ – theoretical values; y – modeled values; N – number of data; e – visibility prediction error.

**Section S2**

***Monte Carlo (Iman Conover)***

The IC method is an algorithm commonly used in the Monte Carlo method. The method can be appropriately used when the following conditions are met (Wu and Tsang 2004):

a) mean values (μ_1_, μ_2_, …, μ_i_)_s_ and standard deviations (σ_1_, σ_2_, …, σ_i_)_s_ of individual variables in the predicted rainfall data series do not differ more than ε < 5% from relevant values of the theoretical distributions, where ε is the relative difference between the modelled and measured value,

b) the empirical distributions of the modelled variable values (x_i_) are in line with theoretical distributions; for the verification of this condition, it is recommended to use the Kolmogorov-Smirnov test,

c) the correlation coefficient (R) between individual variables (x_i_) obtained for data received from the MC simulation does not differ more than about ε_c_.

Normalised input, and output variables were calculated according to the equation:

$x_{i}^{*}=\frac{x_{i}-x_{i,min}}{x_{i,max}-x_{i,min}}$ (4S)

${Vis}^{*}=\frac{Vis-{Vis}_{min}}{{Vis}_{max}-{Vis}_{min}}$ (5S)

where: x_i_ – input data values, x_i,min_ - minimum value of i - this input data, x_i,max_ - maximum value of i - this input data, Vis - visibility value, Vis_min_ - minimum visibility value obtained from measurements, Vis_max_ - maximum visibility value obtained from measurements.


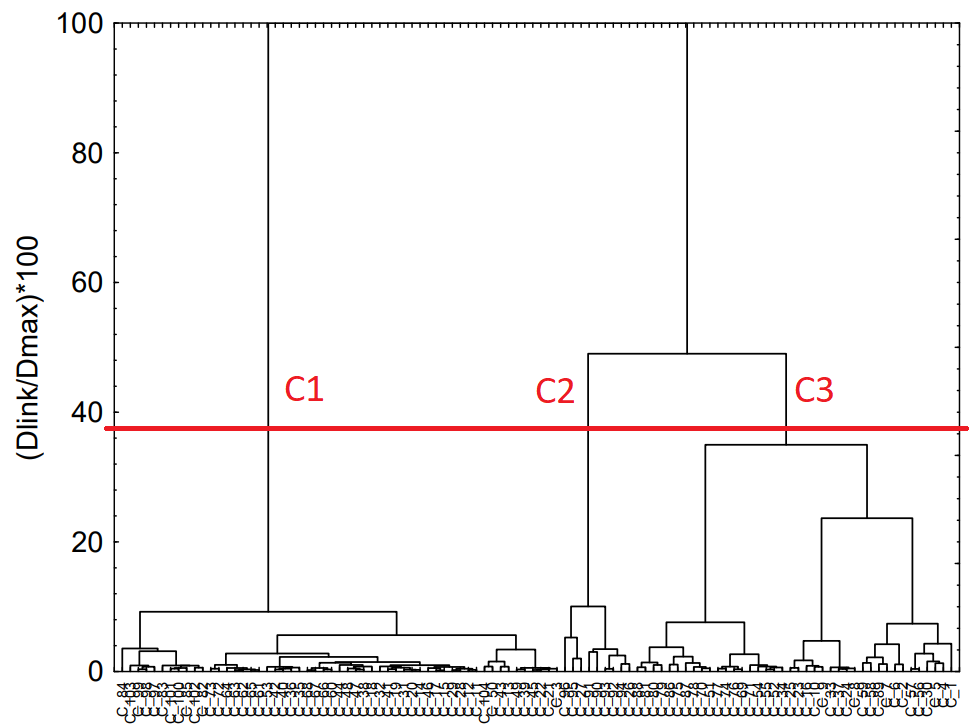


**Figure S1.** Data dendrogram including measurements of meteorological data, air quality and elemental composition for Zabrze (where: Dlink/Dmax – normalised distance between clusters)


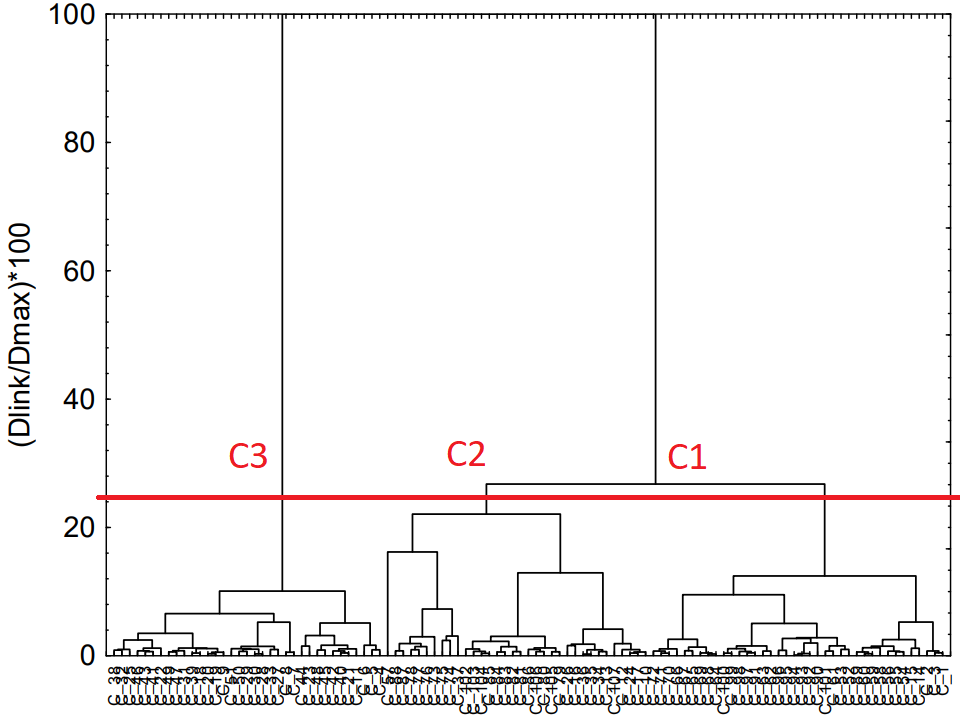


**Figure S2.** Data dendrogram including measurements of meteorological data, air quality and elemental composition for Warsaw


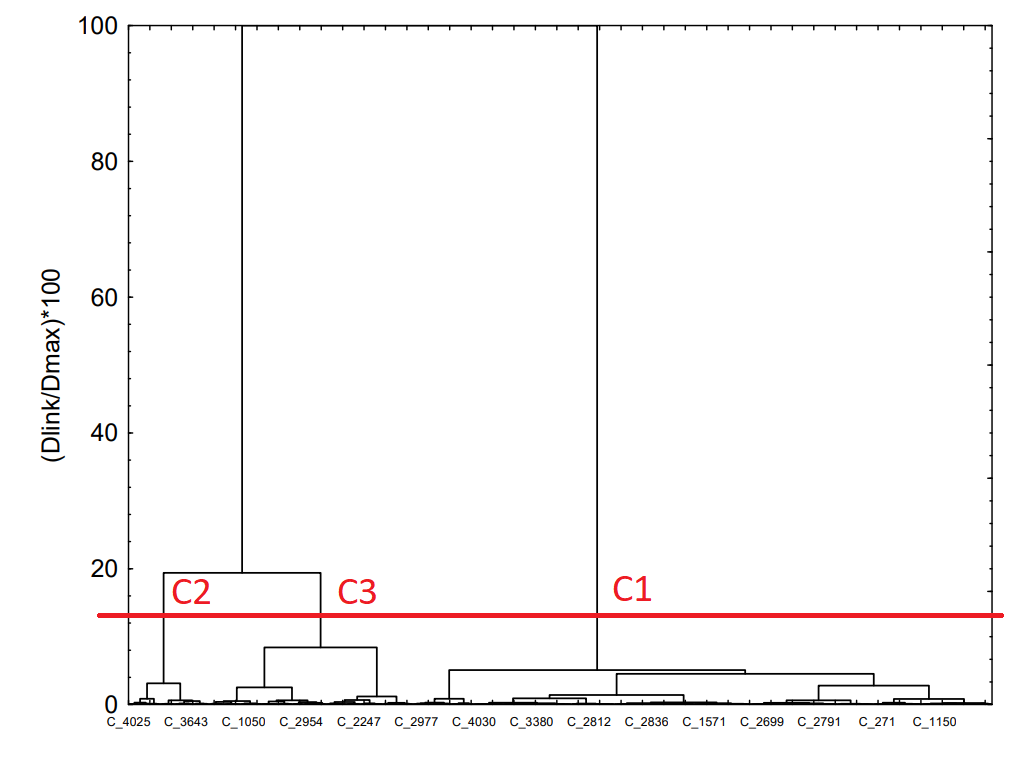


**Figure S3.** Data dendrogram including MC simulations of meteorological data, air quality and elemental composition for Zabrze


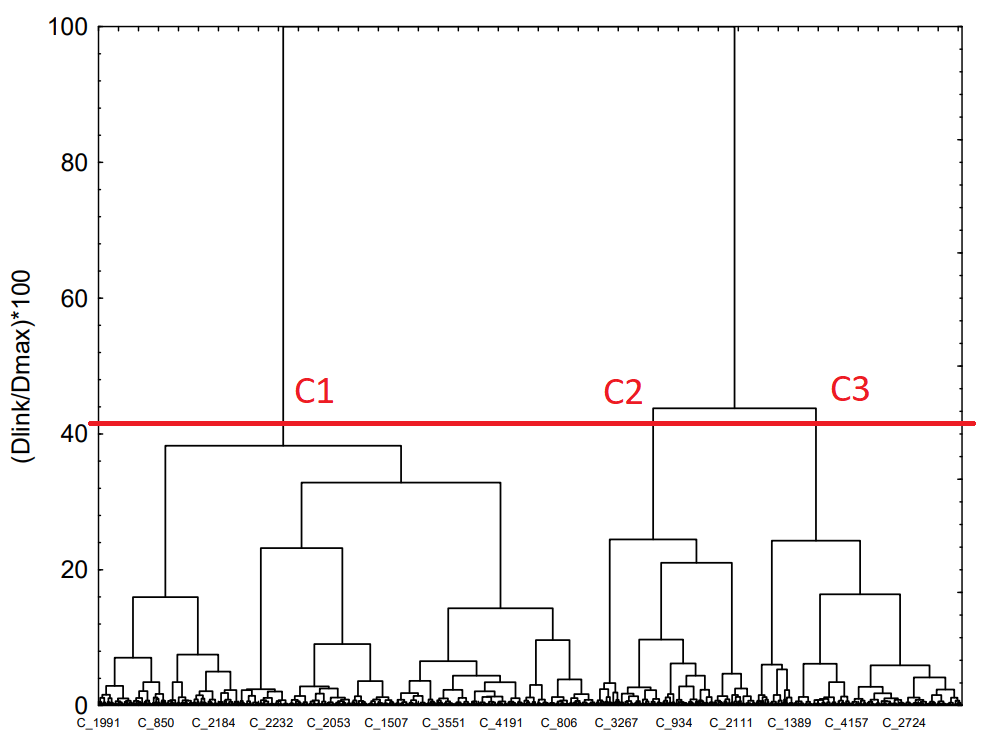


**Figure S4.** Data dendrogram including MC simulations of meteorological data, air quality and elemental composition for Warszawa

**Table S1.** Results test – p for Fischer – Snedecor test

| Warszawa | | Zabrze | |
| --- | --- | --- | --- |
| Variables | p - test | Variables | p - test |
| T | 0.000000 | T | 0.000000 |
| H | 0.000000 | PM1 | 0.000000 |
| Prec | 0.000000 | H | 0.000000 |
| PM1 | 0.000005 | Pb | 0.000000 |
| Ti | 0.000050 | Mo | 0.000001 |
| Zn | 0.000075 | Vi | 0.000010 |
| Pb | 0.000306 | Ti | 0.000021 |
| Cr | 0.002111 | Cd | 0.000856 |
| Mo | 0.002235 | As | 0.001365 |
| Cd | 0.012254 | Cr | 0.002441 |
| Rb | 0.019117 | Al | 0.026591 |
| As | 0.040102 | Prec | 0.034000 |

**Table S2** Structure and Input Variables of MLP Models with Different Input Variants

| Variant | L.neuron | function activation |
| --- | --- | --- |
|  | learning | |
| Zabrze | | |
| main | 8 | tanh |
| main+direction wind | 8 | tanh |
| full | 12 | tanh |
| Warsaw | | |
| main | 7 | tanh |
| main+direction wind | 8 | tanh |
| full | 15 | tanh |

**Table S3** The mean values of selected measurement data for clusters CL1, CL2, CL3 for Warsaw

| Cluster | T | H | Prec | PM1 | Vis | Ti | Zn | Pb | Cr | Mo | Cd | As | Rb |
| --- | --- | --- | --- | --- | --- | --- | --- | --- | --- | --- | --- | --- | --- |
| CL1 | 19.92 | 65.38 | 2.59 | 11.00 | 11.44 | 57.22 | 10.99 | 4.93 | 24.52 | 22.03 | 0.23 | 2.66 | 1.07 |
| CL2 | 2.66 | 81.21 | 1.49 | 12.46 | 8.12 | 8.07 | 20.20 | 6.48 | 14.96 | 29.85 | 0.21 | 1.96 | 0.39 |
| CL3 | 1.36 | 74.36 | 0.52 | 26.10 | 7.02 | 15.33 | 32.80 | 14.12 | 18.13 | 28.36 | 0.42 | 1.91 | 0.61 |

**Table S4** The mean values of selected measurement data for clusters CL1, CL2, CL3 for Zabrze

| Cluster | T | H | Prec | PM1 | Vis | Pb | Mo | Vi | Ti | Cd | As | Cr | Al. |
| --- | --- | --- | --- | --- | --- | --- | --- | --- | --- | --- | --- | --- | --- |
| CL1 | 18.70 | 78.37 | 3.81 | 12.17 | 9.58 | 11.41 | 17.32 | 7.70 | 42.51 | 0.50 | 2.33 | 19.46 | 87.70 |
| CL2 | 2.35 | 81.60 | 2.27 | 33.74 | 7.76 | 16.33 | 3.36 | 0.78 | 2.14 | 0.45 | 2.43 | 5.94 | 167.05 |
| CL3 | 1.20 | 88.71 | 1.13 | 60.51 | 4.71 | 32.33 | 3.66 | 1.06 | 14.76 | 0.91 | 4.00 | 7.00 | 278.92 |

**Table S5.** Theoretical distribution and results of tests (KS, Chi-sq, AD) for independent variables (Warsaw)

| Variable | Distribution | | KS | Chi-sq | AD | Coefficients |
| --- | --- | --- | --- | --- | --- | --- |
| PM1 | GEV | | 0.982 | 0.384 | 0.998 | 0.707;0.180;0.093;0.293;0.534;0.213 |
| Vi | John | | 0.109 | 0.053 | 0.176 | 0.310;0.040;0.022;0.690;0.259;0.158 |
| Zn | GEV | | 0.368 | 0.056 | 0.376 | 0.861;0.058;0.032;0.139;0.258;0.221 |
| As | GEV | | 0.966 | 0.881 | 0.862 | 0.248;0.134;-0.086 |
| Rb | GEV | | 0.879 | 0.313 | 0.764 | 0.081;0.057;0.214 |
| Cd | GEV | | 0.985 | 0.939 | 0.603 | 0.131;0.099;0.277 |
| Pb | GEV | | 0.873 | 0.984 | 0.481 | 0.110;0.089;0.293 |
| Cr | GEV | | 0.16 | 0.052 | 0.4 | 0.114;0.092;0.248 |
| Ti | mix | | 0.23 | 0.052 | 0.321 | 0.317;0.0004;0.005;0.085;0.484;0.263 |
| T | GEV | | 0.902 | 0.075 | 0.881 | 0.446;0.353;-0.598 |
| H | GEV | | 0.747 | 0.231 | 0.058 | 0.536;0.227;-0.417 |
| Prec | John | | 0.325 | 0.265 | 0.32 | 3.00;2.136;0.449;1.421;-0.008 |
| Mn | GEV | | 0.406 | 0.320 | 0.378 | 1.464; 0.800; 0.202 |
| Co | | GEV | 0.591 | 0.320 | 0.425 | 0.219; 0.085; 0.309 |
| Ni | | Johnson | 0.072 | 0.056 | 0.0600 | 2.000; 3.274; 1.782; 5.292 |
| Cu | | GEV | 0.986 | 0.322 | 0.521 | 1.426; 0.982; 0.270 |
| Sr | | GEV | 0.232 | 0.121 | 0.200 | 0.793; 0.522; 0.596 |
| Ga | | GEV | 0.063 | 0.055 | 0.059 | 0.158; 0.235; 1.579 |
| Mo | | Johnson | 0.882 | 0.622 | 0.750 | 2.000; 0.655; 1.816; 13.508 |
| Al | | GEV | 0.144 | 0.056 | 0.102 | 8.621; 15.499; 1.554 |
| Mg | | GEV | 0.592 | 0.315 | 0.420 | 35.585; 32.278; 1.100 |
| OC | | GEV | 0.986 | 0.425 | 0.520 | 3.102; 1.599; 0.426 |
| EC | | GEV | 0.949 | 0.520 | 0.600 | 0.891; 0.406; 0.081 |
| w | | GEV | 0.657 | 0.312 | 0.400 | 2.191; 0.743; 0.177 |

**Table S6.** Theoretical distributions and results of tests (KS, Chi-sq, AD) for independent variables (Zabrze)

| Variable | Distribution | | KS | Chi-sq | AD | | Coefficients | |
| --- | --- | --- | --- | --- | --- | --- | --- | --- |
| PM1 | GEV | | 0.082 | 0.052 | 0.262 | | 0.043; 0.050; 0.824 | |
| Vi | GEV | | 0.213 | 0.064 | 0.136 | | 0.035; 0.048; 1.080 | |
| As | GEV | | 0.988 | 0.764 | 0.977 | | 0.196; 0.128; 0.024 | |
| Cd | GEV | | 0.946 | 0.927 | 0.973 | | 0.099; 0.083; 0.249 | |
| Pb | GEV | | 0.742 | 0.611 | 0.871 | | 0.086; 0.068; 0.316 | |
| Cr | GEV | | 0.897 | 0.228 | 0.578 | | 0.045; 0.048; 0.701 | |
| Mo | GEV | | 0.524 | 0.062 | 0.469 | | 0.073; 0.106; 0.938 | |
| Al | John | | 0.288 | 0.082 | 0.325 | | 3.000; 1.834; 0.723; 1.341; -0.037 | |
| Ti | John | | 0.423 | 0.096 | 0.381 | | 3.000; 1.306; 1.089; 1.551; -0.161 | |
| T | Mix | | 0.793 | 0.396 | 0.847 | | 0.519; 0.192; 0.094; 0.481; 0.814; 0.110 | |
| H | GEV | | 0.892 | 0.142 | 0.832 | | 0.616; 0.238; -0.587 | |
| Prec | mix | | 0.052 | 0.054 | 0.052 | | 0.694; 0.025; 0.051; 0.305; 0.231; 0.227 | |
| Mn | GEV | | 0.904 | 0.339 | 0.389 | | 3.682; 2.051; 0.316 | |
| Co | | GEV | 0.971 | 0.370 | | 0.421 | 0.183; 0.062; 0.130 |  |
| Ni | | Weibull | 0.132 | 0.089 | | 0.124 | 42.66; 0.954 |  |
| Cu | | Jonhson | 0.892 | 0.190 | | 0.224 | 3.000; 1.924; 1.105; 0.945 |  |
| Sr | | GEV | 0.751 | 0.058 | | 0.129 | 0.812; 0.373; 0.296 |  |
| Ga | | Weibull | 0.520 | 0.321 | | 0.412 | 0.455; 0.062; 0.336 |  |
| Mo | | Weibull | 0.595 | 0.062 | | 0.106 | 8.602; 0.717 |  |
| Al | | GEV | 0.389 | 0.122 | | 0.216 | 18.85; 31.22; 1.61 |  |
| Mg | | GEV | 0.064 | 0.051 | | 0.045 | 47.417; 41.444; 1.005 |  |
| OC | | GEV | 0.252 | 0.054 | | 0.189 | 4.160; 3.022; 0.958 |  |
| EC | | Johnson | 0.345 | 0.123 | | 0.216 | 3.000; 3.541; 1.172; 38.362 |  |

**Table S7.** Comparison of regression model coefficients between Zabrze and Warsaw

| Zabrze | | | | Warszawa | | | |
| --- | --- | --- | --- | --- | --- | --- | --- |
| Input | Value | St.der | p-test | Input | Value | St.der | p-test |
| Intercept | 0.520 | 0.013 | 0.00000 | Intercept | 0.860 | 0.011 | 0.00000 |
| PM | 0.063 | 0.032 | 0.02583 | PM | 0.597 | 0.017 | 0.00000 |
| Vi | 0.163 | 0.051 | 0.00231 | Vi | 0.040 | 0.049 | 0.04015 |
| Mn | 0.051 | 0.008 | 0.00000 | Mn | 0.074 | 0.014 | 0.00000 |
| Co | 0.231 | 0.011 | 0.00000 | Co | 0.383 | 0.014 | 0.00000 |
| Ni | 0.014 | 0.010 | 0.03174 | Ni | 0.197 | 0.009 | 0.00000 |
| Cu | 0.044 | 0.009 | 0.00001 | Cu | 0.196 | 0.015 | 0.00000 |
| Zn | 0.070 | 0.015 | 0.00002 | Zn | 0.027 | 0.008 | 0.00128 |
| As | 0.037 | 0.020 | 0.03543 | As | 0.087 | 0.013 | 0.00000 |
| Rb | 0.017 | 0.034 | 0.03614 | Rb | 0.253 | 0.012 | 0.00000 |
| Sr | 0.228 | 0.012 | 0.00000 | Sr | 0.041 | 0.013 | 0.00261 |
| Cd | 0.018 | 0.011 | 0.10275 | Cd | 0.089 | 0.008 | 0.00000 |
| Pb | 0.064 | 0.018 | 0.00062 | Pb | 0.170 | 0.008 | 0.00000 |
| Ga | 0.194 | 0.033 | 0.00000 | Ga | 0.044 | 0.015 | 0.00526 |
| Cr | 0.111 | 0.057 | 0.04557 | Cr | 0.047 | 0.048 | 0.03291 |
| Mo | 0.087 | 0.009 | 0.00000 | Mo | 0.123 | 0.011 | 0.00000 |
| Al | 0.104 | 0.011 | 0.00000 | Al | 0.336 | 0.005 | 0.00000 |
| Mg | 0.043 | 0.009 | 0.00001 | Mg | 0.012 | 0.006 | 0.03983 |
| Ti | 0.268 | 0.009 | 0.00000 | Ti | 0.080 | 0.006 | 0.00000 |
| OC | 0.068 | 0.038 | 0.03831 | OC | 0.212 | 0.022 | 0.00000 |
| EC | 0.173 | 0.022 | 0.00000 | EC | 0.014 | 0.011 | 0.03899 |
| T | 0.421 | 0.012 | 0.00000 | T | 0.271 | 0.009 | 0.00000 |
| Rh | 0.352 | 0.008 | 0.00000 | Rh | 0.420 | 0.006 | 0.00000 |
| Prec | 0.144 | 0.010 | 0.00000 | Prec | 0.077 | 0.005 | 0.00000 |
| w | 0.226 | 0.010 | 0.00000 | w | 0.200 | 0.007 | 0.00000 |
| R2=0.998 | | | | R2=0.999 | | | |

**Table S8.** Correlations between air quality parameters, meteorological conditions and elements

|  | PM1 | Vis | Vi | Mn | Co | Ni | Cu | Zn | As | Rb | Sr | Cd | Pb | Ga | Cr | Mo | Al | Mg | Ti | OC | EC | T | H | Prec | V |
| --- | --- | --- | --- | --- | --- | --- | --- | --- | --- | --- | --- | --- | --- | --- | --- | --- | --- | --- | --- | --- | --- | --- | --- | --- | --- |
| PM1 | 1,00 | -0,50 | -0,28 | 0,58 | -0,01 | 0,02 | 0,24 | 0,66 | -0,06 | 0,00 | -0,12 | 0,60 | 0,68 | -0,09 | -0,12 | 0,27 | 0,10 | 0,00 | -0,27 | **0,90** | 0,72 | -0,32 | 0,14 | -0,30 | -0,19 |
| Vis | -0,50 | 1,00 | 0,31 | -0,29 | 0,12 | -0,06 | -0,07 | -0,49 | 0,10 | 0,36 | 0,22 | -0,31 | -0,43 | 0,20 | 0,02 | -0,40 | -0,15 | -0,04 | 0,43 | -0,50 | -0,52 | 0,71 | -0,69 | -0,01 | -0,07 |
| Vi | -0,28 | 0,31 | 1,00 | -0,29 | 0,30 | 0,12 | 0,20 | -0,31 | 0,71 | 0,54 | 0,13 | -0,14 | -0,15 | 0,40 | 0,88 | -0,11 | 0,13 | 0,34 | 0,80 | -0,31 | -0,32 | 0,42 | -0,20 | 0,07 | -0,21 |
| Mn | 0,58 | -0,29 | -0,29 | 1,00 | 0,21 | 0,50 | 0,35 | 0,59 | -0,12 | -0,07 | 0,20 | 0,52 | 0,61 | -0,06 | -0,18 | 0,39 | 0,12 | 0,05 | -0,28 | 0,63 | 0,47 | -0,20 | 0,02 | -0,14 | 0,11 |
| Co | -0,01 | 0,12 | 0,30 | 0,21 | 1,00 | 0,25 | 0,39 | -0,01 | 0,22 | 0,34 | 0,27 | 0,25 | 0,08 | 0,28 | 0,23 | 0,04 | 0,02 | 0,11 | 0,52 | -0,06 | -0,07 | 0,30 | -0,20 | -0,07 | -0,12 |
| Ni | 0,02 | -0,06 | 0,12 | 0,50 | 0,25 | 1,00 | 0,16 | 0,01 | 0,32 | 0,05 | 0,09 | 0,13 | 0,12 | 0,16 | 0,21 | 0,37 | 0,16 | 0,15 | 0,09 | 0,10 | 0,03 | -0,03 | 0,15 | 0,09 | 0,14 |
| Cu | 0,24 | -0,07 | 0,20 | 0,35 | 0,39 | 0,16 | 1,00 | 0,32 | 0,18 | 0,22 | 0,26 | 0,26 | 0,34 | 0,18 | 0,17 | 0,31 | 0,07 | 0,40 | 0,31 | 0,19 | 0,24 | 0,06 | -0,01 | 0,09 | -0,21 |
| Zn | 0,66 | -0,49 | -0,31 | 0,59 | -0,01 | 0,01 | 0,32 | 1,00 | -0,07 | -0,11 | 0,14 | 0,68 | 0,79 | 0,00 | -0,05 | 0,25 | -0,02 | -0,01 | -0,37 | 0,73 | 0,74 | -0,51 | 0,24 | -0,14 | -0,03 |
| As | -0,06 | 0,10 | 0,71 | -0,12 | 0,22 | 0,32 | 0,18 | -0,07 | 1,00 | 0,60 | 0,12 | -0,06 | 0,01 | 0,60 | 0,79 | 0,19 | 0,08 | 0,36 | 0,59 | -0,05 | -0,10 | 0,18 | 0,02 | 0,01 | -0,18 |
| Rb | 0,00 | 0,36 | 0,54 | -0,07 | 0,34 | 0,05 | 0,22 | -0,11 | 0,60 | 1,00 | 0,30 | 0,07 | 0,00 | 0,68 | 0,45 | 0,04 | 0,06 | 0,33 | 0,64 | -0,10 | -0,15 | 0,54 | -0,31 | -0,10 | -0,41 |
| Sr | -0,12 | 0,22 | 0,13 | 0,20 | 0,27 | 0,09 | 0,26 | 0,14 | 0,12 | 0,30 | 1,00 | 0,16 | 0,06 | 0,53 | 0,12 | -0,04 | -0,07 | 0,08 | 0,21 | -0,14 | -0,11 | 0,21 | -0,20 | 0,05 | -0,12 |
| Cd | 0,60 | -0,31 | -0,14 | 0,52 | 0,25 | 0,13 | 0,26 | 0,68 | -0,06 | 0,07 | 0,16 | 1,00 | 0,75 | 0,14 | 0,02 | 0,13 | -0,01 | -0,10 | -0,11 | 0,64 | 0,60 | -0,29 | -0,04 | -0,32 | -0,09 |
| Pb | 0,68 | -0,43 | -0,15 | 0,61 | 0,08 | 0,12 | 0,34 | 0,79 | 0,01 | 0,00 | 0,06 | 0,75 | 1,00 | -0,01 | 0,08 | 0,29 | 0,09 | 0,05 | -0,19 | 0,78 | 0,71 | -0,46 | 0,11 | -0,24 | 0,05 |
| Ga | -0,09 | 0,20 | 0,40 | -0,06 | 0,28 | 0,16 | 0,18 | 0,00 | 0,60 | 0,68 | 0,53 | 0,14 | -0,01 | 1,00 | 0,45 | -0,04 | -0,04 | 0,15 | 0,45 | -0,17 | -0,09 | 0,30 | -0,13 | 0,00 | -0,30 |
| Cr | -0,12 | 0,02 | 0,88 | -0,18 | 0,23 | 0,21 | 0,17 | -0,05 | 0,79 | 0,45 | 0,12 | 0,02 | 0,08 | 0,45 | 1,00 | 0,03 | 0,15 | 0,37 | 0,60 | -0,08 | -0,10 | 0,06 | 0,03 | 0,06 | -0,10 |
| Mo | 0,27 | -0,40 | -0,11 | 0,39 | 0,04 | 0,37 | 0,31 | 0,25 | 0,19 | 0,04 | -0,04 | 0,13 | 0,29 | -0,04 | 0,03 | 1,00 | 0,26 | 0,39 | -0,07 | 0,32 | 0,30 | -0,32 | 0,27 | 0,04 | 0,06 |
| Al | 0,10 | -0,15 | 0,13 | 0,12 | 0,02 | 0,16 | 0,07 | -0,02 | 0,08 | 0,06 | -0,07 | -0,01 | 0,09 | -0,04 | 0,15 | 0,26 | 1,00 | 0,45 | 0,12 | 0,11 | -0,01 | -0,10 | 0,04 | 0,00 | 0,13 |
| Mg | 0,00 | -0,04 | 0,34 | 0,05 | 0,11 | 0,15 | 0,40 | -0,01 | 0,36 | 0,33 | 0,08 | -0,10 | 0,05 | 0,15 | 0,37 | 0,39 | 0,45 | 1,00 | 0,29 | 0,04 | -0,01 | 0,07 | 0,03 | 0,14 | -0,08 |
| Ti | -0,27 | 0,43 | 0,80 | -0,28 | 0,52 | 0,09 | 0,31 | -0,37 | 0,59 | 0,64 | 0,21 | -0,11 | -0,19 | 0,45 | 0,60 | -0,07 | 0,12 | 0,29 | 1,00 | -0,35 | -0,38 | 0,58 | -0,34 | -0,02 | -0,36 |
| OC | 0,90 | -0,50 | -0,31 | 0,63 | -0,06 | 0,10 | 0,19 | 0,73 | -0,05 | -0,10 | -0,14 | 0,64 | 0,78 | -0,17 | -0,08 | 0,32 | 0,11 | 0,04 | -0,35 | 1,00 | 0,81 | -0,46 | 0,13 | -0,30 | -0,06 |
| EC | 0,72 | -0,52 | -0,32 | 0,47 | -0,07 | 0,03 | 0,24 | 0,74 | -0,10 | -0,15 | -0,11 | 0,60 | 0,71 | -0,09 | -0,10 | 0,30 | -0,01 | -0,01 | -0,38 | 0,81 | 1,00 | -0,51 | 0,19 | -0,24 | -0,14 |
| T | -0,32 | 0,71 | 0,42 | -0,20 | 0,30 | -0,03 | 0,06 | -0,51 | 0,18 | 0,54 | 0,21 | -0,29 | -0,46 | 0,30 | 0,06 | -0,32 | -0,10 | 0,07 | 0,58 | -0,46 | -0,51 | 1,00 | -0,59 | -0,04 | -0,39 |
| H | 0,14 | -0,69 | -0,20 | 0,02 | -0,20 | 0,15 | -0,01 | 0,24 | 0,02 | -0,31 | -0,20 | -0,04 | 0,11 | -0,13 | 0,03 | 0,27 | 0,04 | 0,03 | -0,34 | 0,13 | 0,19 | -0,59 | 1,00 | 0,24 | 0,06 |
| Prec | -0,30 | -0,01 | 0,07 | -0,14 | -0,07 | 0,09 | 0,09 | -0,14 | 0,01 | -0,10 | 0,05 | -0,32 | -0,24 | 0,00 | 0,06 | 0,04 | 0,00 | 0,14 | -0,02 | -0,30 | -0,24 | -0,04 | 0,24 | 1,00 | 0,11 |
| V | -0,19 | -0,07 | -0,21 | 0,11 | -0,12 | 0,14 | -0,21 | -0,03 | -0,18 | -0,41 | -0,12 | -0,09 | 0,05 | -0,30 | -0,10 | 0,06 | 0,13 | -0,08 | -0,36 | -0,06 | -0,14 | -0,39 | 0,06 | 0,11 | 1,00 |
